# Exosomal transfer of miR‐106a‐5p contributes to cisplatin resistance and tumorigenesis in nasopharyngeal carcinoma

**DOI:** 10.1111/jcmm.16801

**Published:** 2021-09-01

**Authors:** Jiaxing Li, Chaoquan Hu, Hui Chao, Yu Zhang, Yong Li, Jing Hou, Limin Huang

**Affiliations:** ^1^ Guizhou university medical college Guiyang China; ^2^ Department of Surgery Affiliated Hospital GuiZhou Medical University Guiyang China; ^3^ Department of Oncology Guizhou Cancer Center Guizhou Provincial People's Hospital Guiyang China

**Keywords:** ARNT2, cisplatin, exosomes, miR‐106a‐5p, nasopharyngeal carcinoma

## Abstract

Nasopharyngeal carcinoma (NPC), a subclass of cancers of the neck and head, is a predominant cause of cancer‐associated death worldwide. Hence, there is a critical need for research into NPC‐related treatment strategies. Cisplatin is a promising therapy option for NPCs and other cancers that is frequently utilized. Some patients acquire resistance to cisplatin therapy, which complicates the successful use of cisplatin treatment in NPCs. Although exosomal transfer of oncogenic miRNAs has been shown to improve recipient cell proliferation, metastasis and chemoresistance, the molecular mechanism behind this effect on NPC has yet to be fully understood. Exosomal microRNAs (miRNAs) from cisplatin‐resistant cells were identified as significant mediators of chemoresistance in NPC cells in this investigation. Initially, we found that exosomal miR‐106a‐5p levels in the serum of chemoresistant and last‐cycle patients were greater than in that of non‐resistant and first‐cycle patients. Also, exosomal miR‐106a‐5p enhanced the proliferative ability of NPC cells. Mechanistically, exosomal miR‐106a‐5p targets ARNT2, which further activates AKT phosphorylation, and thus promotes NPC cell proliferation, decreases apoptosis and in turn regulates tumorigenesis. We found similar results using in vivo NPC models, where exosomal miR‐106a‐5p through regulation of ARNT2 (aryl hydrocarbon receptor nuclear translocator 2) promoted tumorigenesis. Taken together, these findings indicate that exosomal miR‐106a‐5p could be a promising diagnostic biomarker and drug target for patients with NPC.

## 1 INTRODUCTION

Nasopharyngeal carcinoma (NPC) is one of the most prevalent cancers affecting the neck and head regions, specifically among North African, South Asian, Chinese, Alaskan and the Middle Eastern populations.^1^, ^2^, ^3^ Previous research has identified EBV infection, genetic susceptibility and other environmental factors as potential causes of NPC.^4^ To date, treatment strategies vary from radiotherapy for early stages to a combination of radiochemotherapy for later stages of NPC. These treatment strategies have allowed 84%–90% of 5‐year survival rate for early‐stage NPC, but up to 20% of five‐year survival rate for late‐stage NPC.^5^ One of the commonly used chemotherapeutic treatment strategies for NPC and many other cancers is cisplatin.^6^ However, there are rising concerns associated with the development of resistance against cisplatin specifically among NPC patients, which minimizes the effect of cisplatin in subsequent rounds of chemotherapeutic treatment.^7^, ^8^ However, the mechanism underlying the development of this resistance is still unknown.

Extracellular vesicles such as exosomes are released by the cells as a vital form of carrier that transports information from one cell to another.^9^, ^10^ Exosomes are lipid bilayer molecules that can transport protein, messenger RNA, small non‐coding RNA and other molecules from cells such as malignant cells, macrophages and dendritic cells.^11^, ^12^, ^13^, ^14^ With a size range of 40–100 nm, exosomes from malignant cells have been identified to promote tumorigenesis by upregulating proliferation, metastasis and angiogenesis.^13^, ^15^ Exosomes have been found to mediate the transmission of malignancy from affected cells to healthy cells, thereby increasing proliferation, metastasis and angiogenesis in the tumour microenvironment.^16^, ^17^ Recently, more studies have identified that indeed the exosomes carry molecules that enable cellular resistance to certain drugs.^18^, ^19^ However, there needs to be more detailed understanding of the mechanisms contributing to this resistance.

MicroRNAs (miRs) are a class of non‐coding RNAs that have been identified to confer tumorigenesis.^20^ These miRs are usually 20–22 nt in length and play a vital role in the regulation of target genes. Exosomes are important carriers of these miRs, and they are transported to achieve specific functions in specific cells.^17^ Many studies have shown that the main function of miR is to control tumour progression, and one such miR is miR‐106a‐5p.^21^, ^22^ A study on ovarian cancer identified miR‐106a‐5p could promote tumour progression by binding and regulating the target gene ARHGAP24.^21^ Another study identified that indeed miR‐106a‐5p was highly expressed and associated with a poor prognosis in triple‐negative breast cancer.^22^ Studies on gastric cancer^23^ and hepatocellular carcinoma^24^ also confirmed that miR‐106a‐5p indeed played a vital role in tumorigenesis.

Recently, studies have identified that indeed these miRs carried by exosomes play a key role in conferring resistance to chemotherapeutic treatments. Exosomal miR‐744 was found to confer resistance to sorafenib treatment in hepatocellular carcinoma.^19^ Interestingly, exosomes transferred from resistant cell lines carried over the resistance to sensitive lines. In cervical cancer, the lncRNA HNF1A‐AS1 contributed to the drug resistance against cisplatin.^25^ The aim of this study was to determine the role of exosomal miR‐106a‐5p in conferring resistance in NPC using both *in vitro* and *in vivo* models.

## 2 MATERIALS AND METHODS

### 2.1 Patients and samples

Patient tissue samples were obtained from NPC patients who underwent tumour resection at the Medical College of Guizhou University. Tumour and healthy tissue samples were collected and stored at −80°C until use. Additionally, serum samples were collected from NPC patients and processed to collect exosomes before they underwent surgery. Additionally, the samples were thoroughly centrifuged to separate the cell debris. Serum and exosomal samples from patients and healthy controls were stored until further use at −80°C. The study was approved by the Medical College of Guizhou University's ethical committee, and written informed consent was obtained from all patients.

### 2.2 Cell culture

NPC cell line CNE1 was obtained from the Medical College of Guizhou University. Cells were cultured in Dulbecco's modified Eagle's medium (DMEM, Gibco) with 10% foetal bovine serum (FBS, Gibco) and 1% penicillin/streptomycin (P/S, Gibco). The cells were then cultured at 37°C with 5% CO_2_. To create a cisplatin‐resistant line (CNE1r), CNE1 cells were treated with increasing concentrations of cisplatin, starting with 0.5 µM and increased by doubling every two weeks until the concentration of cisplatin reached 8 µM. To keep the resistance in the CNE1r cells, the cells were treated with 8 µM of cisplatin biweekly.

### 2.3 Fluorescence in situ hybridization

The paraffin‐embedded tissues were fixed with formalin, and dual‐labelled with digoxin (DIG) and LNA‐enhanced miR‐106a‐5p probe (miRCURY LNA miRNA detection probe, Qiagen) in situ hybridization to detect miRNA. The probe was denatured at 83℃ for 5 mins. Tissue sections were heated in an oven at 65℃ for 2 h, then denatured and dehydrated in sodium citrate saline (SCC). The denatured probe (40 nM) was mixed with the tissue section and incubated overnight at 37℃. The slides were washed extensively and then incubated with anti‐DIG fluorescein isothiocyanate (FITC)‐conjugated antibody for 1 h at 37℃, washed again, drained and used 4'‐6'diamino‐2‐benzindole (DAPI) counterstain. The LNA‐FISH signal was observed with a fluorescence microscope (TE‐2000E, Nikon).

### 2.4 Exosomal isolation

Initially, cells were cultured in 15‐cm dishes containing 30 ml of culture medium. When the cells reached 70% confluence, they were thoroughly washed with PBS and cultured with DMEM for 48 h containing 10% FBS, which was exosome‐depleted. Finally, the supernatant was collected by ultracentrifugation at 100,000 *g* for 12 h.

### 2.5 Exosomal preparation

Exosomal release was assessed by detecting exosomal markers such as CD81 and HSP70 using Western blot analysis and flow cytometry. Cell culture media were used to obtain exosomes. Exosomes were prepared using standard differential centrifugation in the following manner: centrifugation at 3000 × *g* for 20 min at 4℃ to obtain plasma, then 10,000 × *g* for 20 min at 4℃ to remove cells and platelets, and then twice at 100,000 × *g* for 70 min at 4℃ with a SW‐41 rotor, followed by washes with phosphate‐buffered saline (PBS). PBS was used solely for vehicle control. A total of 5 µg of exosomes was incubated for 15 mins with 1.25 µl aldehyde/sulphate latex beads, 4% w/v (4 µm, A37304; Invitrogen), and then incubated with anti‐CD81 (ab219209; Abcam) and anti‐Hsp70 (ab183435; Abcam). After fixing with 1% PFA, flow cytometry was performed with a FACSCalibur flow cytometer (BD Biosciences). The results were analysed by a CytoFLEX instrument (Beckman Coulter).

### 2.6 Transmission Electron Microscope

The isolated exosomes were further characterized using Transmission Electron Microscope (TEM) based on a previously published protocol.^19^ Exosomes were fixed with 1% glutaraldehyde for 10 min and then transferred onto carbon‐coated copper grids. Further, the exosomes were stained with 1% uranyl acetate and dried. Finally, the exosomes were imaged using HT700 TEM (Hitachi).

### 2.7 Cell viability (CCK‐8) assay

Cell counting kit −8 (CCK‐8) was used to assess the cell viability based on the manufacturer's instruction. Briefly, cells were seeded into a 48‐well plate and treated with cisplatin. After 24 h, the culture media with the drug were replaced with fresh media containing 10 μl of CCK‐8. After incubation for 2 h, absorbance at 450 nm was read.

### 2.8 Colony formation assay

The cells were seeded into a 6‐well plate and allowed to grow. At the end of 14 days, the colonies formed were fixed and stained with 0.1% crystal violet staining solution. The stained colonies were imaged and counted.

### 2.9 Cell apoptosis analysis

Cells were initially detached with 0.25% trypsin and thoroughly washed with cold PBS. Following that, the cells were stained with annexin V/fluorescein isothiocyanate (FITC) and propidium iodide (PI) based on the manufacturer's instructions. The fluorescence of FITC and PI was determined at 488 nm through 515 or 620 nm using a fluorescence‐activated cell sorter, and the cell apoptosis was assessed.

### 2.10 Histological analysis

Tumour samples or cells were fixed with paraformaldehyde and washed thoroughly before processing. Tumour samples were embedded on an OCT and cryosectioned at 10 µM thickness and stained. Sections were further stained with haematoxylin and eosin (Sigma). Cells were stained with respective primary antibodies and secondary antibodies as mentioned in a previous study.^18^ Finally, tissues and cells were visualized and imaged under a microscope.

### 2.11 Exosomal labelling and uptake

Staining of exosomes was performed using PKH26 dye. Further, the cells were then cultured with the labelled exosomes for 3 h. The cells were finally fixed and nuclear‐stained with DAPI. The stained cells were then imaged for further quantification of exosomal uptake.

### 2.12 RNA extraction and quantitative reverse transcription‐PCR (RT‐qPCR)

The total RNA was isolated from the exosomes using the miRNeasy Micro Kit (QIAGEN) based on the manufacturer's instructions. Furthermore, total RNA was extracted from patient samples and the cell lines using TRIzol reagent (Invitrogen, USA) based on the manufacturer's instructions. To assess the exosomal miR‐106a‐5p expression levels, we used Hairpin‐it micro‐RNA quantification kit and followed the manufacturer's instructions. And for tissue or cell samples, we used TaqMan MicroRNA Assays (Applied Biosystems) and followed the manufacturer's instructions. All the primers used in this experiment are as follows: for ARNT2—forward, 5'‐ACC AGCGAGACGGGCTGTCA‐3' and reverse, 5'‐GTGCCCGGC AGGGAATGGAC‐3'; and for GAPDH—forward, 5'‐GGGCTCTCTGCTCCTCCCTCT‐3' and reverse, 5'‐CCGTTGAAC TTGCCGTGGGT‐3'.

### 2.13 Western blot analysis

Either the cells or the exosomes were lysed using RIPA lysis buffer with protease inhibitor (Genestar Biotechnology). Further, the extracted protein was quantified and 20 µg of protein was loaded onto 10%–20% SDS‐PAGE and migrated for 1 h. Further, the gel was blotted and transferred onto a nitrocellulose membrane. The membrane was then blocked using 5% skim milk for 1 h and incubated with primary antibodies (anti‐ARNT2, anti‐Hsp70, anti‐CD81, anti‐cleaved caspase‐3 antibodies and β‐actin) in skim milk overnight at 4℃. After a thorough wash, the membrane was incubated with secondary antibody for 1 h. The membrane was then visualized and imaged using chemiluminescence reagents.

### 2.14 Luciferase assay

To test luciferase activity, we inserted either a 3′ wild‐type UTR or a 3′ mutant UTR of ARNT2, which is the target site of miR‐106a‐5p onto a psi‐Check‐2 vector. We then transfected the CNE1 cells with psi‐Check‐2 vector containing either the ARNT2 3′‐WT‐UTR or the 3′‐MUT‐UTR in the presence or absence of miR‐106a‐5p inhibitor. The luciferase activity was finally assessed using the Dual‐Luciferase Reporter Assay System after 48 h.

### 2.15 Migration and invasion assay

Transwell migration assays (Millipore) were used to assess migration and invasion capacity of CNE1 cells, as described previously. Initially, cells were seeded into serum‐free media onto the upper chamber and placed on dishes with overgrowth medium and 10% FBS. To assess invasion, we pre‐coated the upper chamber with Matrigel. Further, the cells that invaded the Matrigel were stained with crystal violet, imaged and counted after 48 h.

### 2.16 Cell transfection

All plasmids containing either miR‐106a‐5p inhibitor or mimics and their respective controls were purchased from Genewiz. The cells were transfected with these plasmids using Lipofectamine 2000 (Invitrogen) based on the manufacturer's instructions.

### 2.17 TUNEL staining

CNE1 cells were fixed with paraformaldehyde for 15 mins and washed thrice with PBS. The cells were stained with terminal deoxynucleotidyl transferase dUTP nick‐end labelling (TUNEL) kit (Beyotime) based on the manufacturer's instructions. The nucleus was stained with DAPI, and imaging was done using an Olympus microscope (Olympus).

### 2.18 Xenograft studies and treatment experiments

All animal experiments performed in this study were approved by the Animal Care and Use Committee of the Medical College of Guizhou University. Mice were housed in our animal facility and were allowed to feed and drink ad libitum. Mice were then grouped into 4 groups with 6 mice in each group. Further, the mice in each group were injected subcutaneously with 0.5 × 10^5^ CNE1r + PBS, CNE1s + PBS, CNE1s + rExo, and CNE1r + 106‐5p‐down rExo cells, respectively. All mice were further injected with 4 mg/kg/day of cisplatin for 15 days. Through the 15 days, the volume of the xenografts was measured. At the end of 15th day, the mice were killed and the xenograft tumours were excised, and further analysis was done.

### 2.19 Statistical analysis

Statistical analysis was performed using SPSS v.17.0 software (SPSS). All the results are presented as mean ±  SD. Statistical analysis for two groups was performed using Student's two‐tailed *t* test, whereas for multiple groups, one‐way analysis of variance (ANOVA) was used. *p* < 0.05 was considered statistically significant.

## 3 RESULTS

### 3.1 Upregulation of exosomal miR‐106a‐5p during the course of cisplatin‐based chemotherapy

**FIGURE 1:**
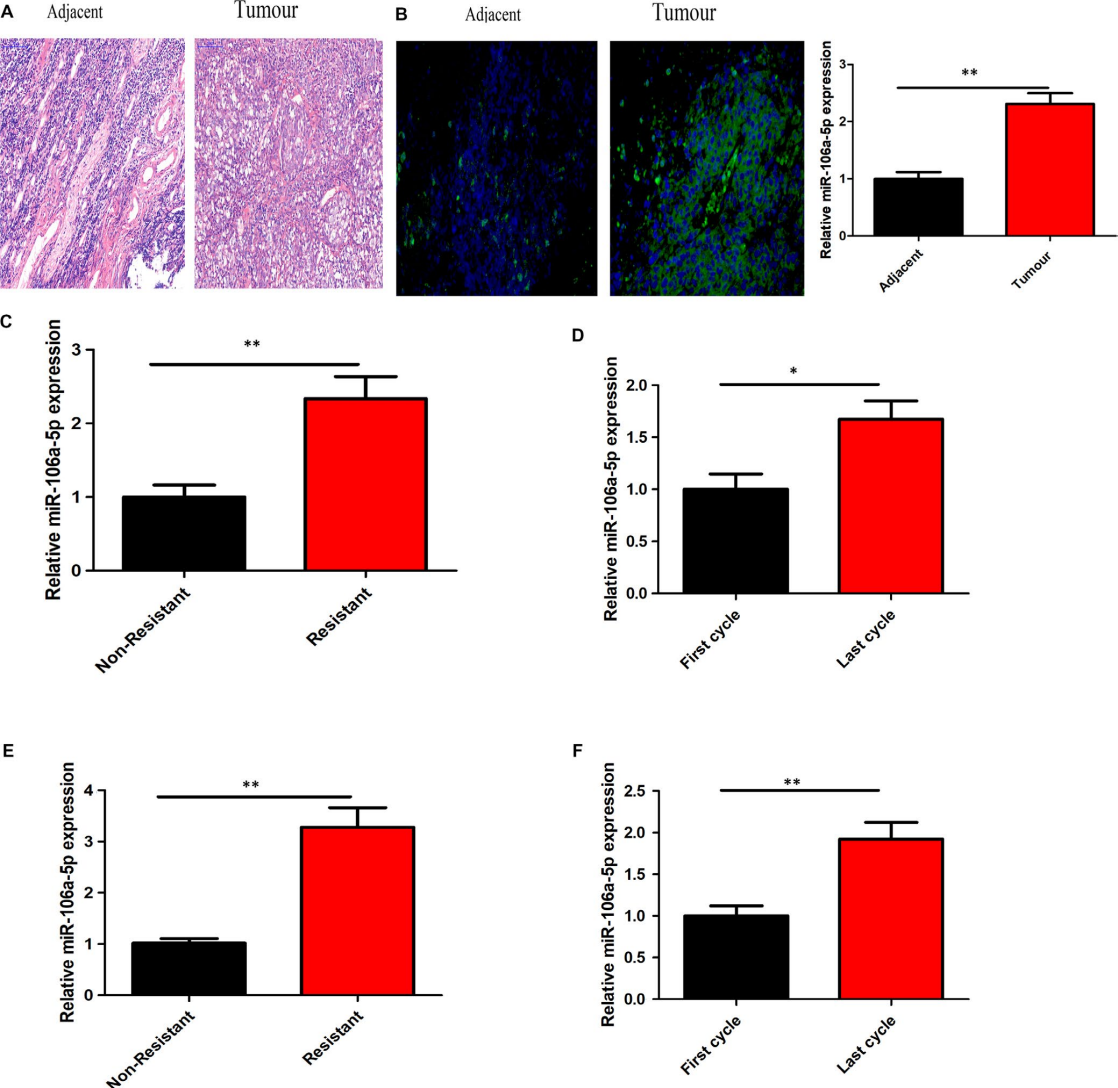
Upregulation of exosomal miR‐106a‐5p during the course of cisplatin‐based chemotherapy. (A) Haematoxylin and eosin staining of the tumour and adjacent tissues in NPC patients. The level of miR‐106a‐5p in tumour and adjacent tissues of NPC patients was measured by FISH (B) and RT‐PCR (C). (D) Serum samples of NPC patients are collected (A) at the start of treatment (non‐resistant) and the day of disease progression (resistant) or (E) at the start of the first cycle and last cycle of treatment among NPC patients before being diagnosed as resistant. The level of miR‐106a‐5p in serum was evaluated by qRT‐PCR. Circulating exosomes were isolated from paired serum samples of NPC patients (F) non‐resistant and resistant or (G) of NPC patients receiving the first and last cycle of treatment before diagnosed as drug‐resistant. The level of miR‐106a‐5p in exosomes was evaluated by qRT‐PCR. In all experiments, bars represent mean  ±  SD for three replicates. *p < 0.05 and **p < 0.01

Examining the GEO database (GEO login number: GSE70970), as shown in Figure S1 A and B, the expression of miR‐106a‐5p was significantly increased among miRNAs differentially expressed in nasopharyngeal carcinoma tissues. To determine the role of miR‐106a‐5p in NPC, we first stained tumour tissue samples with haematoxylin and eosin, which revealed clear cell disorganization when compared to adjacent healthy tissue samples (Figure 1 A). FISH was used to confirm the expression of miR‐106a‐5p in NPC tumour tissue. We discovered that miR‐106a‐5p was overexpressed in NPC tumour tissues (Figure 1 B). Additionally, we observed that miR‐106a‐5p expression levels significantly increased in NPC tumour samples compared with those in the adjacent tissue samples (Figure 1 C, n = 13). Further, we examined paired serum samples from NPC patients at the start of cisplatin‐based chemotherapy (non‐resistant) and the day of progression of the disease (resistant), and observed that miR‐106a‐5p levels were significantly increased among the resistant group than those among the non‐resistant (Figure 1 D, *n* = 19). We also collected paired serum samples from NPC patients before being diagnosed as resistant, at the first and last cycle of cisplatin‐based chemotherapy. We observed that miR‐106a‐5p levels were significantly increased in serum samples obtained after the last cycle of chemotherapy than those obtained after the first cycle (Figure 1 E, *n* = 15). Next, we derived the circulating exosomes from paired resistant and non‐resistant serum samples of NPC patients undergoing cisplatin‐based chemotherapy and observed that miR‐106a‐5p levels were significantly increased in the circulating exosomes of resistant patients (Figure 1 F, *n* = 19). Subsequently, we also assessed the levels of miR‐106a‐5p in circulating exosomes during the first and last cycles of cisplatin‐based chemotherapy. We observed that miR‐106a‐5p expression levels were significantly increased during the last cycle of the cisplatin‐based chemotherapy (Figure 1 G, *n* = 15).

### 3.2 miR‐106a‐5p is enriched along with an increased number of exosomes in cisplatin‐resistant CNE1 cells

**FIGURE 2:**
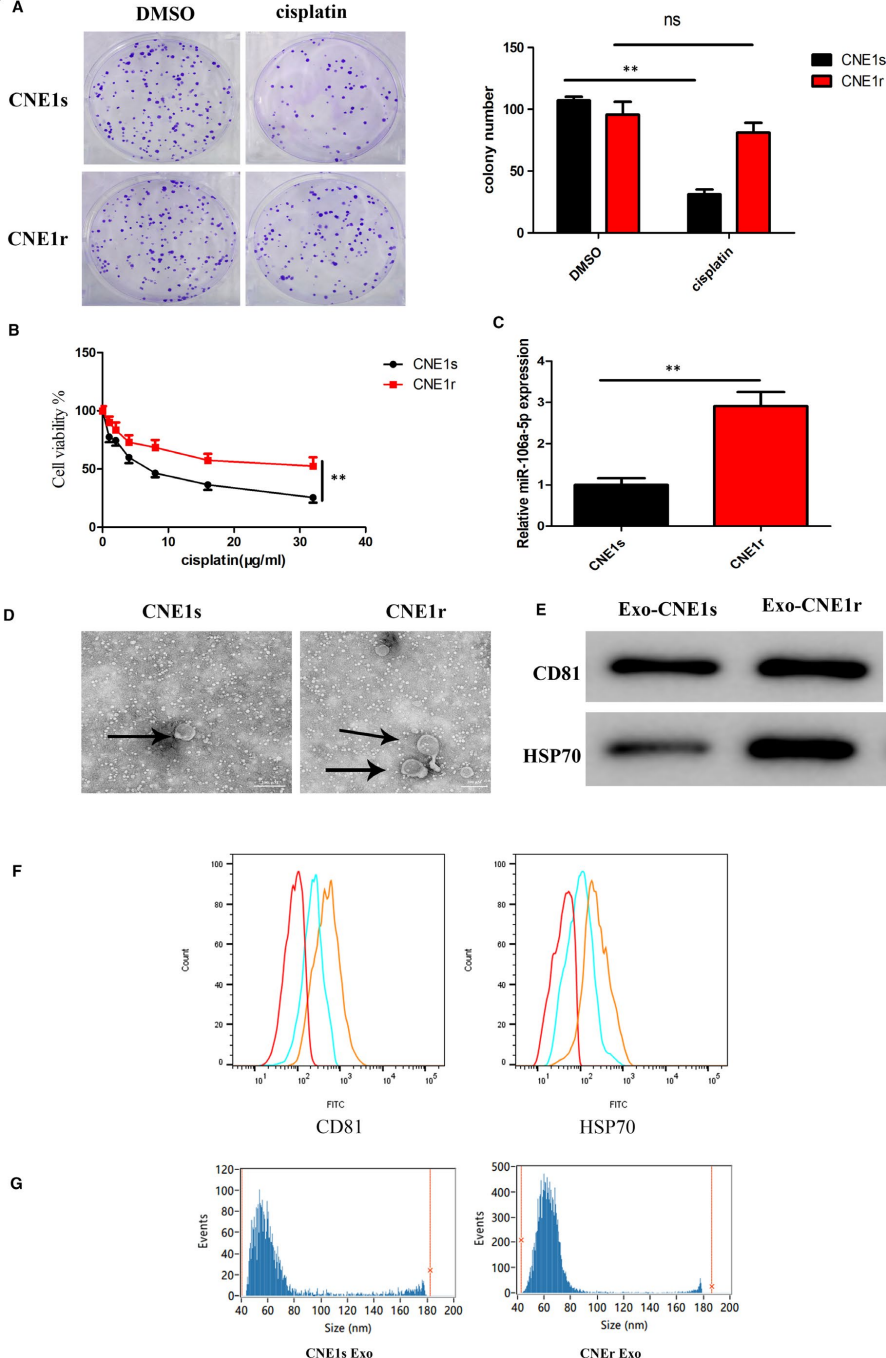
miR‐106a‐5p is enriched along with an increased number of exosomes in cisplatin‐resistant CNE1 cells. (A) Plate colony formation assays were used to estimate the cisplatin resistance of CNE1r cells. (B) Relative cell viability of CNE1s and CNE1r cells with cisplatin treatment(0, 1, 2, 4, 8, 16 and 32 μg/ml) for 48 h. (C) Relative expression of miR‐106a‐5p in CNE1s and CNE1r was measured by RT‐qPCR. (D) Transmission electron microscopic images of exosomes derived from CNE1s and CNE1r. (E) Exosomal‐positive markers CD81 and HSP70 were detected in CNE1s‐derived and CNE1r‐derived exosomes using Western blotting. (F) Flow cytometry of the exosomal surface marker CD81 and HSP70 level. (G) The size distribution of the isolated exosomes was measured by high‐sensitivity flow cytometry. CNE1s, cisplatin‐sensitive CNE1 cells; CNE1r, cisplatin‐resistant CNE1 cells. In all experiments, bars represent mean ± SD for three replicates. *p <  0.05 and **p < 0.01

To assess the role of miR‐106a‐5p in cisplatin resistance, we first constructed both the sensitivity and the resistance model of CNE1 and CNE2 cells, the IC50 and RI of these cell lines for cisplatin are also shown in Table 1. The results showed that CNE1r was more resistant to cisplatin than CNE2r cells, so next, we used an *in vitro* model of CNE1 NPC cell line. Initially, we developed a CNE1 cisplatin‐resistant variant, which is referred to as CNE1r cell. Further, we confirmed the cisplatin resistance in these cells using colony formation assay. On treatment with cisplatin, there were significantly lower colonies formed by CNE1‐susceptible (CNE1s) cells. However, there were no significant differences between the colonies formed by CNE1r and control‐untreated cells, confirming the cisplatin resistance in CNE1r cells (Figure 2 A). We also performed a cell viability assay and observed that indeed CNE1r cells were highly viable compared with CNE1s cells when treated with cisplatin (Figure 2B). Interestingly, we observed that miR‐106a‐5p expression levels were significantly higher among CNE1r cells (Figure 2C). Further, we extracted exosomes from CNE1r and CNE1s cells and assessed their morphological characteristics using transmission electron microscopy (TEM) (Figure 2D). Exosomes isolated from CNE1r and CNE1s were positive for CD81 and HSP70 by Western blot and flow cytometry. The results showed an increased expression level of both CD81 and HSP70, indicating an increased level of exosomes in CNE1r cells (Figure 2E). Simultaneously, FACS analysis of the surface proteins CD81 and HSP70 revealed that they were present in both CNE1s and CNE1r Exo. The fluorescence expression of CD81 and HSP70 in exosomes isolated from CNE1r cells was higher after normalization to control beads compared with the CNE1s cell group. (Figure 2F). High‐sensitivity flow cytometric analysis of the exosomes indicated that the number of exosomes was significantly higher in the CNE1r cells than in the CNE1s cells (Figure 2G).

**TABLE 1:** IC50 and RI of cell lines CNE1, CNE2, CNE1DDP and CNE2DDP

Cell line	IC50 (mg/L)	RI
CNE1	0.91 ± 0.38	4.97
CNE1r	4.53 ± 0.24*	
CNE2	5.98 ± 0.44	2.88
CNE2r	17.23 ± 0.56*	

Abbreviation: RI, resistance index.

* represents *p* < 0.05, compared with CNE1 or CNE2, as determined using Student's *t* test.

### 3.3 miR‐106a‐5p is enriched in cisplatin‐resistant cell‐derived exosomes and conferred cisplatin resistance to NPC via exosomal transfer

**FIGURE 3:**
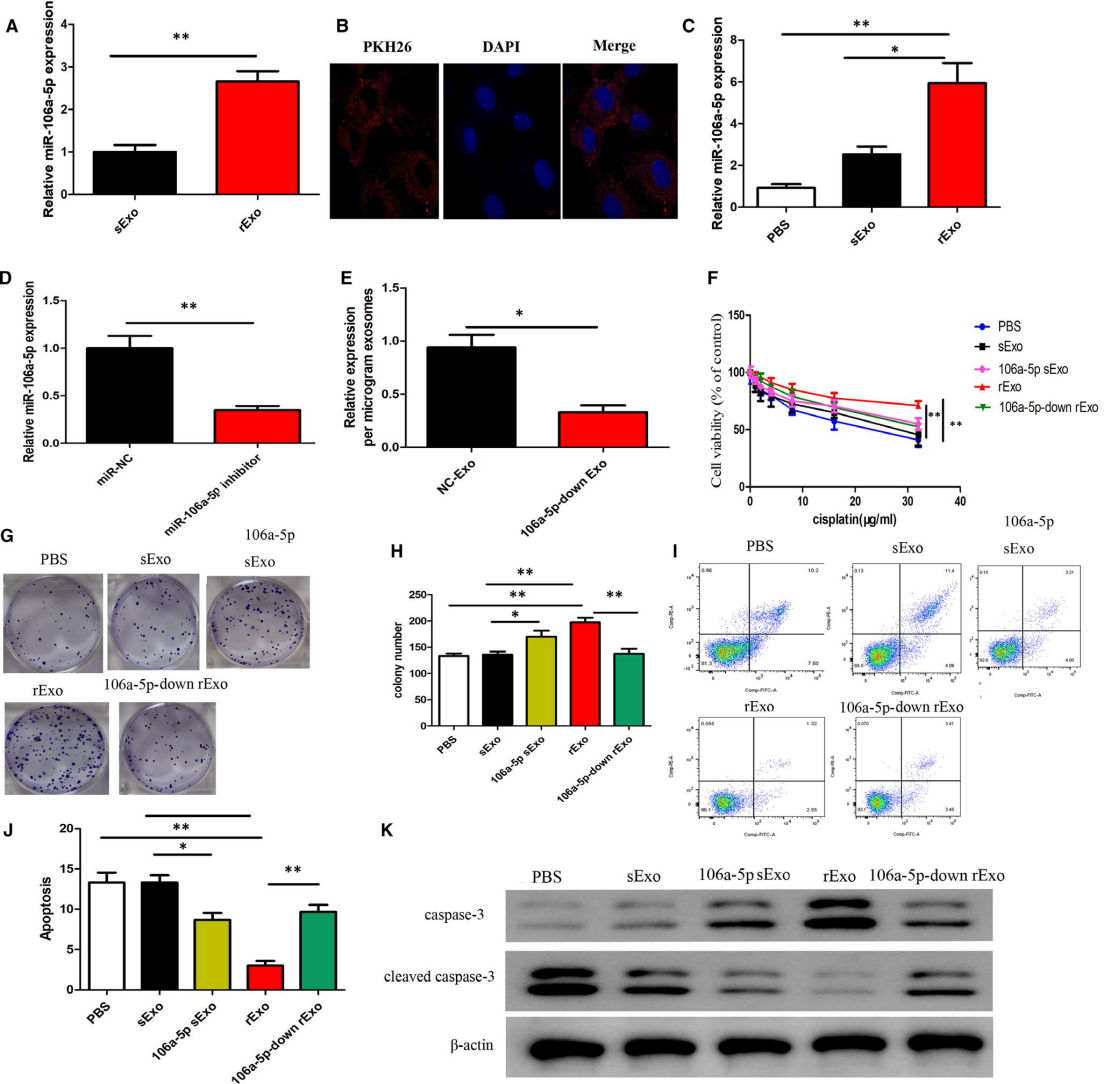
miR‐106a‐5p is enriched in cisplatin‐resistant cell‐derived exosomes and conferred cisplatin resistance to NPC via exosomal transfer. (A) qRT‐PCR of miR‐106a‐5p in rExo and sExo groups. (B) Confocal microscopy showed exosomal internalization by CNE1 cells after incubation with PKH26‐labelled (red fluorescence) rExo. DAPI was used to stain the nuclei of CNE1 recipient cells with blue fluorescence. Scale bar = 25 µm. (C) Level of miR‐106a‐5p in exosome‐treated CNE1‐sensitive cells compared with control PBS‐treated cells. (D) Relative expression of miR‐106a‐5p levels after transfection of miR‐NC or miR‐106a‐5p inhibitor in CNE1r cells. (E) Relative expression of miR‐106a‐5p levels of NC exosomes and 106a‐5p‐down exosomes. (F) Relative cell viability of cisplatin‐sensitive cells, which were pre‐treated with different exosomes (30 μg/ml) for 48 h under cisplatin treatment for indicated concentrations. (G) Plate colony formation assays of the CNE1s after treatment with different exosomes (30 μg/ml) with cisplatin treatment. (H) Quantitative analysis of the colony formation assay performed in (G). (I) FACS analysis was performed to assess the apoptotic rates of CNE1s fed with different exosomal treatments (30 μg/ml) with 16 μg/ml cisplatin for 48 h. (J) Quantitative statistical analysis of results from (I). (K) Levels of apoptotic protein cleaved caspase‐3 in CNE1s after treatment with different exosomes (30 μg/ml) with 16 μg/ml cisplatin for 48 h. CNE1s, cisplatin‐sensitive CNE1 cells; CNE1r, cisplatin‐resistant CNE1 cells. In all experiments, bars represent mean ± SD for three replicates. *p < 0.05 and **p < 0.01

Following that, we next isolated exosomes from both CNE1r and CNE1s, hence referred to as rExo and sExo, respectively. We observed a significantly high level of miR‐106a‐5p among the rExo group than that among the sExo group (Figure 3A). Further, we cultured the CNE1s with media containing the rExo (labelled with PKH26) and using confocal microscopy confirmed the internalization of these rExo cells into the CNE1s (Figure 3B). Interestingly, we observed that CNE1s cultured with rExo had an increased expression level of miR‐106a‐5p compared with CNE1s cultured with sExo (Figure 3C). These results indicate the potential transfer of miR‐106a‐5p from resistant CNE1 cells to sensitive CNE1s through exosomes. Next, we inhibited miR‐106a‐5p in CNE1r cells and confirmed its efficacy using qRT‐PCR (Figure 3D). Indeed, miR‐106a‐5p levels were significantly decreased in CNE1r cells treated with miR‐106a‐5p inhibitor compared with miR‐NC. We isolated the exosomes from these cells and observed that inhibition of miR‐106a‐5p significantly decreased the level of miR‐106a‐5p levels relative to the exosomal levels; that is, in a constant microgram level of exosomes, miR‐106a‐5p levels were significantly decreased. These data indicated that in the presence of miR‐106a‐5p inhibitor, the levels of miR‐106a‐5p carried by the exosomes were significantly decreased compared with the controls (Figure 3E). In the presence of varying concentrations of cisplatin, we tested the cell viability of CNE1‐sensitive cells cultured with exosomes from CNE1s (sExo), CNE1r (rExo), CNE1s treated with miR‐106a‐5p mimics (106a‐5p sExo), or CNE1r treated with miR‐106a‐5p inhibitor (106a‐5p‐down rExo). Initially, it was clear that CNE1s cells gained resistance to cisplatin when cultured with rExo, indicating a potential transfer of resistance from resistant CNE1 to susceptible CNE1 through the exosomes. However, it was clear that cisplatin could significantly decrease the viability of cells treated with 106a‐5p‐down rExo compared with rExo. These data indicated that indeed downregulation of miR‐106a‐5p could significantly decrease the effect of rExo in conferring resistance to cisplatin treatment (Figure 3F). We also checked using colony formation assay that indeed treatment with 106a‐5p‐down rExo could significantly decrease the number of colonies formed when compared to the cells treated with rExo in the presence of cisplatin treatment (Figure 3G, H). Next, we performed flow cytometric analysis to assess the level of apoptosis. Initially, it was clear that CNE1s cultured with sExo and treated with cisplatin had high levels of cell death. However, cells cultured with rExo and treated with cisplatin had very low levels of cell death, confirming the resistance to cisplatin treatment. Interestingly, cells treated with rExo containing downregulated miR‐106a‐5p showed an increase in cell death, confirming that indeed downregulation of miR‐106a‐5p is vital for decreasing the resistance to cisplatin (Figure 3I, J). To further assess the role of miR‐106a‐5p in cisplatin resistance, we checked the levels of key apoptotic marker cleaved caspase‐3. We observed that the level of cleaved caspase‐3 was significantly decreased in cells cultured with rExo. However, the cleaved caspase‐3 levels were recovered in the cells treated with rExo downregulated for miR‐106a‐5p (Figure 3 K). These results indicated that miR‐106a‐5p carried by the exosomes plays a vital role in conferring cisplatin resistance, and miR‐106a‐5p mimics promoted cell resistance; however, inhibition of miR‐106a‐5p could significantly rescue this resistance by decreasing the cell viability and colony formation, and increasing apoptosis in NPC cells.

### 3.4 ARNT2 is a direct target of miR‐106a‐5p

**FIGURE 4:**
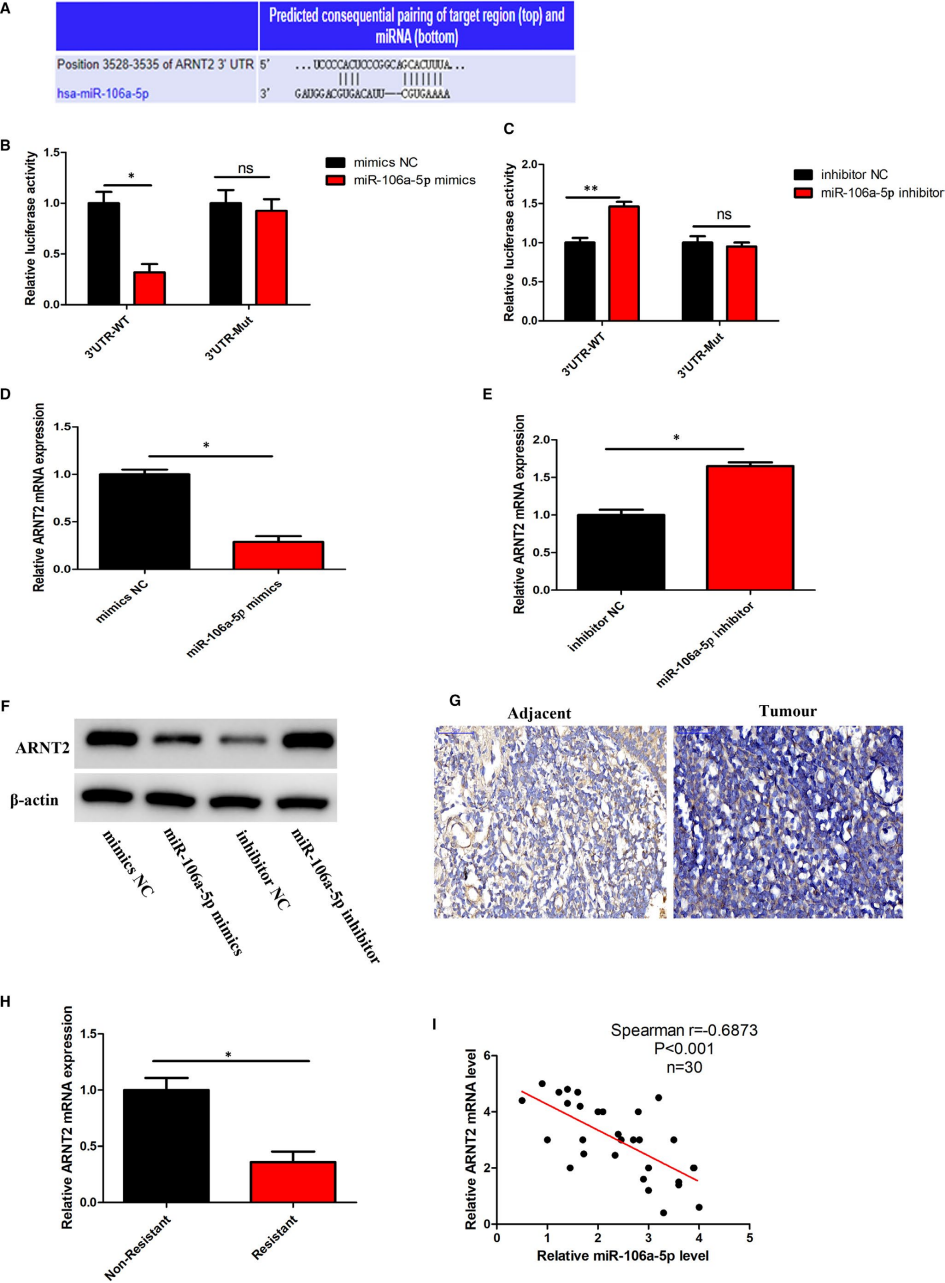
ARNT2 is a direct target of miR‐106a‐5p. (A) The predicted consequential pairing of ARNT2 target region and miR‐106a‐5p. (B) Luciferase activity was analysed in CNE1r cells co‐transfected with miR‐106a‐5p mimics and the luciferase reporter plasmid. (C) Luciferase activity was analysed in CNE1r cells co‐transfected with miR‐106a‐5p inhibitor and luciferase reporter plasmids. (D) The mRNA level of ARNT2 in CNE1r cells treated with miR‐106a‐5p mimics was analysed by RT‐qPCR. (E) The mRNA level of ARNT2 in CNE1r cells treated with miR‐106a‐5p inhibitor was analysed by RT‐qPCR. (F) The protein level of ARNT2 in CNE1r treated with miR‐106a‐5p mimics or miR‐106a‐5p inhibitor was analysed by Western blot (G) Immunohistochemistry (IHC) of ARNT2 in tumour and adjacent tissues of NPC patients. (H) RT‐qPCR of ARNT2 in serum samples of NPC patients from non‐resistant and resistant to cisplatin treatment. (I) Pearson's correlation analysis of the relative expression levels of miR‐106a‐5p and ARNT2. CNE1s, cisplatin‐sensitive CNE1 cells; CNE1r, cisplatin‐resistant CNE1 cells. In all experiments, bars represent mean ± SD for three replicates. *p < 0.05 and **p < 0.01

Using prediction mapping, we further identified that miR‐106a‐5p potentially binds and regulates the expression of ARNT2 (Figure 4A). Furthermore, using wild‐type and mutated 3'UTR of ARNT2 with a luciferase reporter, we performed luciferase activity assay in the presence or absence of miR‐106a‐5p mimics. In cells with wild‐type 3'UTR ARNT2, the use of miR‐106a‐5p mimics significantly decreased the luciferase activity. Alternatively, in cells with mutated 3'UTR ARNT2, the use of miR‐106a‐5p mimics did not cause any decrease in luciferase activity (Figure 4B). This experiment was then repeated in the presence of miR‐106a‐5p inhibitors. Evidentially, with wild‐type 3'UTR ARNT2, inhibition of miR‐106a‐5p significantly increased the luciferase activity, and with mutant 3'UTR ARNT2, inhibition of miR‐106a‐5p did not cause any change in luciferase activity (Figure 4C). These results indicated that miR‐106a‐5p binds to the 3'UTR of ARNT2 and regulates its expression. We further assessed the mRNA expression level of ARNT2 and observed that in the presence of miR‐106a‐5p mimics, the expression of ARNT2 was significantly increased (Figure 4D). However, in the presence of miR‐106a‐5p inhibitors, the expression level of ARNT2 was significantly increased (Figure 4E). Using Western blotting, we confirmed that in the presence of miR‐106a‐5p, ARNT2 was downregulated at the protein level, and in the presence of miR‐106a‐5p inhibitors, the reverse could be observed (Figure 4F). In addition, we further verified that miR‐106a‐5p can regulate the expression of ARNT2 in the nucleus (Figure S2 A). Next, using immunohistochemical staining (IHC), we observed that ARNT2 levels were downregulated in tumour tissue compared with the adjacent healthy tissue (Figure 4G). Evidentially, ARNT2 expression levels were significantly lower among serum samples from cisplatin treatment–resistant patients than those among samples from non‐resistant patients (Figure 4H). Through Pearson's correlation analysis, we observed that indeed the expression of ARNT2 is negatively correlated with miR‐106a‐5p expression (Figure 4I). At the same time, we further verified the effect of ARNT2 on HIF1‐α and found ARNT2 inhibited the expression of HIF1‐α (Figure S2B and C). These results clearly confirmed that miR‐106a‐5p binds to the 3’UTR of ARNT2 and downregulates its expression.

### 3.5 miR‐106a‐5p facilitates cisplatin resistance by targeting ARNT2

**FIGURE 5:**
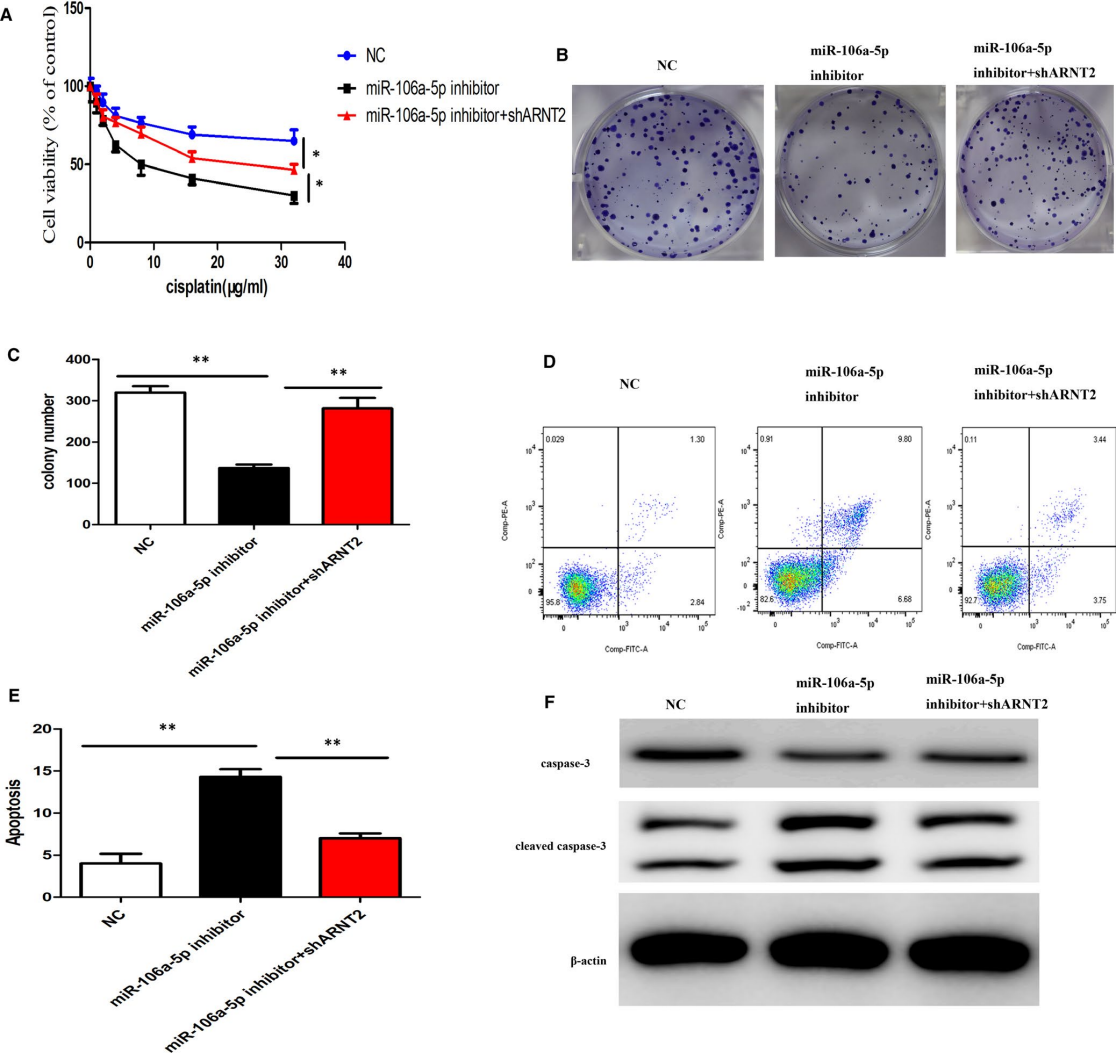
miR‐106a‐5p facilitates cisplatin resistance mainly by targeting ARNT2. (A) Relative cell viability of the NC or miR‐106a‐5p inhibitor‐transfected CNE1r cells with or without shARNT2 transfection under cisplatin treatment for indicated concentrations for 48 h. (B) Plate colony formation assays of the NC or miR‐106a‐5p inhibitor‐transfected CNE1r with or without shARNT2 transfection under 16μg/mL cisplatin treatment. (C) Quantitative analysis of the results from (B). (D) FACS analysis was performed to assess the apoptotic rates of CNE1r transfected with NC, miR‐106a‐5p inhibitor, or miR‐106a‐5p plus shARNT2 with treatment of 16 μg/ml cisplatin for 48 h. (E) Quantitative analysis of results from (D). (F) Western blot analysis of the expression of the apoptotic protein cleaved caspase‐3 in CNE1r cells transfected with NC, miR‐106a‐5p inhibitor, or miR‐106a‐5p inhibitor plus shARNT2 upon treatment with 16 μg/ml cisplatin for 48 h. In all experiments, bars represent mean ± SD for three replicates. *p < 0.05 and **p < 0.01

To assess the mechanistic influence of miR‐106a‐5p on ARNT2, we used CNE1r cells with or without miR‐106a‐5p inhibition; subsequently, these cells were also silenced for ARNT2. Initially, we assessed the cell viability of these cells and observed that inhibition of miR‐106a‐5p significantly decreased the cell viability of CNE1r cells. Interestingly, silencing of ARNT2 recovered the viability in these cells (Figure 5A). Further, we performed a colony formation assay and observed that inhibition of miR‐106a‐5p decreased colony‐forming units; however, the silencing of ARNT2 in these cells significantly recovered the number of colony‐forming units (Figure 5B, C). Next, we assessed the apoptosis levels using flow cytometry and observed that inhibition of miR‐106a‐5p significantly increased apoptosis in CNE1r cells compared with the control. However, the silencing of ARNT2 significantly rescued the cells from apoptosis (Figure 5D, E). Further, we also observed a similar effect in cleaved caspase‐3 levels, where inhibition of miR‐106a‐5p increased cleaved caspase‐3 levels, which were downregulated again when ARNT2 was silenced in these CNE1r cells (Figure 5F). These results indicated that miR‐106a‐5p positively influences tumorigenesis by inhibiting ARNT2, and indeed inhibition of ARNT2 even in the absence of miR‐106a‐5p increased cell viability and colony formation, and decreased apoptosis in CNE1r cells.

### 3.6 Exosomal miR‐106a‐5p targeting of ARNT2 promotes recipient cell proliferation, migration and invasion via Akt phosphorylation

**FIGURE 6:**
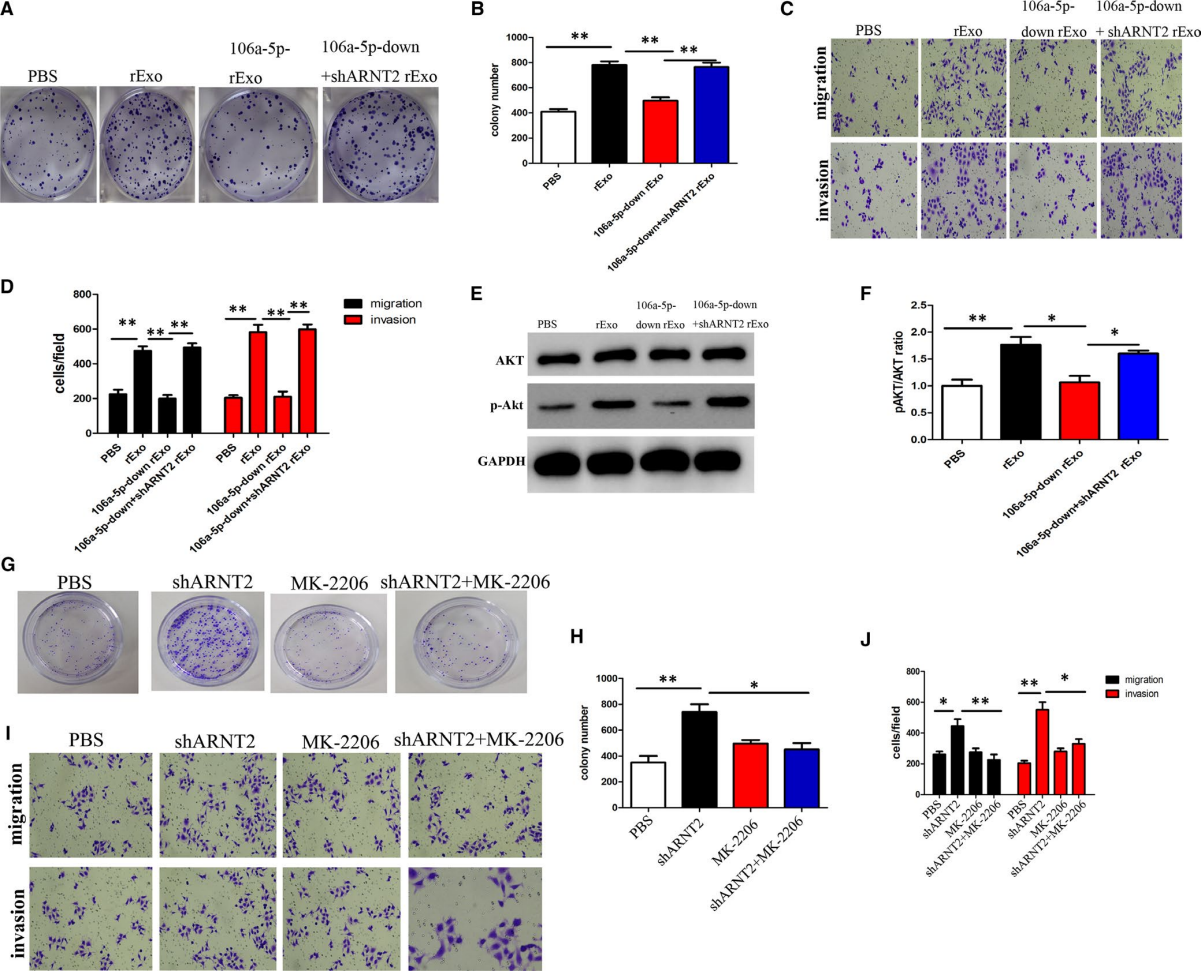
Exosomal miR‐106a‐5p targeting of ARNT2 promotes recipient cell proliferation, migration and invasion via Akt phosphorylation. (A) Plate colony formation assays of the CNE1s after treatment with different exosomes (30 μg/ml). (B) Quantitative statistical analysis of results from (A). (C) Transwell assays of the CNE1s after treatment with different exosomes (30 μg/ml). (D) Quantitative statistical analysis of results from (C). (E) Akt and P‐Akt proteins were tested in CNE1s after treatment with different exosomes (30 μg/ml) by Western blot. (F) Quantitative statistical analysis of results from (E). (G) Plate colony formation assays of the different treated CNE1s. (H) Quantitative statistical analysis of results from (G). (I) Transwell assays of the different treated CNE1s. (J) Quantitative statistical analysis of results from (I). In all experiments, bars represent mean ± SD for three replicates. *p < 0.05 and **p < 0.01

To further assess the effect of exosomal miR‐106a‐5p from CNE1r cells on cisplatin resistance, we initially isolated exosomes from CNE1r cells and cultured them on CNE1s cells in the presence of cisplatin. As previously observed, rExo increased significantly the colony‐forming units of CNE1s cells in the presence of cisplatin, indicating the clear transference of resistance through the exosomes. Furthermore, we cultured CNE1s with exosomes carrying downregulated miR‐106a‐5p and observed that cisplatin could significantly reduce the colony number. Additionally, when we cultured the cells with exosomes from cells downregulated for miR‐106a‐5p and silenced for ARNT2, there was an improvement or rescue in colony‐forming units (Figure 6A, B). We additionally also performed migration and invasion assays and observed that CNE1s cells with rExo had increased migration and invasion capacity compared with control CNE1s in the presence of cisplatin. Interestingly, rExo with downregulated miR‐106a‐5p showed decreased migration and invasion capacity, which could be recovered when ARNT2 was silenced (Figure 6C, D). These data on colony‐forming units, migration and invasion support the hypothesis that exosomal miR‐106a‐5p confers cisplatin resistance and promotes tumorigenesis by downregulating ARNT2 expression. However, we wanted to further explore the molecular mechanisms behind miR‐106a‐5p and ARNT2’s effect on tumorigenesis. To achieve this, we assessed the Akt and p‐Akt levels in CNE1s cells cultured with rExo. We observed that in the presence of rExo, CNE1s possessed an increased ratio of pAKT/AKT levels when compared to control CNE1s. This indeed was clear from the Western blotting results, indicating increased phosphorylation of Akt in CNE1s in the presence of rExo. However, in the presence of downregulated miR‐106a‐5p, the ratio of pAKT/AKT levels significantly decreased, which could be recovered by the silencing of ARNT2 (Figure 6E, F). We hypothesized that since silencing of ARNT2 activated Akt signalling in CNE1 cells, inhibiting Akt signalling could partially reverse the biological effects of ARNT2 silencing. To inhibit Akt signalling, MK‐2206, a highly selective Akt inhibitor was used. MK‐2206 significantly inhibited proliferation in ARNT2‐silenced NPC cells based on cell viability (Figure 6G and H), migration and invasion ability (Figure 6I and J). These findings support the hypothesis that miR‐106a‐5p increases cisplatin resistance and tumorigenesis by regulating ARNT2 and phosphorylation of Akt.

### 3.7 Exosomal miR‐106a‐5p promotes NPC cell cisplatin resistance in vivo

**FIGURE 7:**
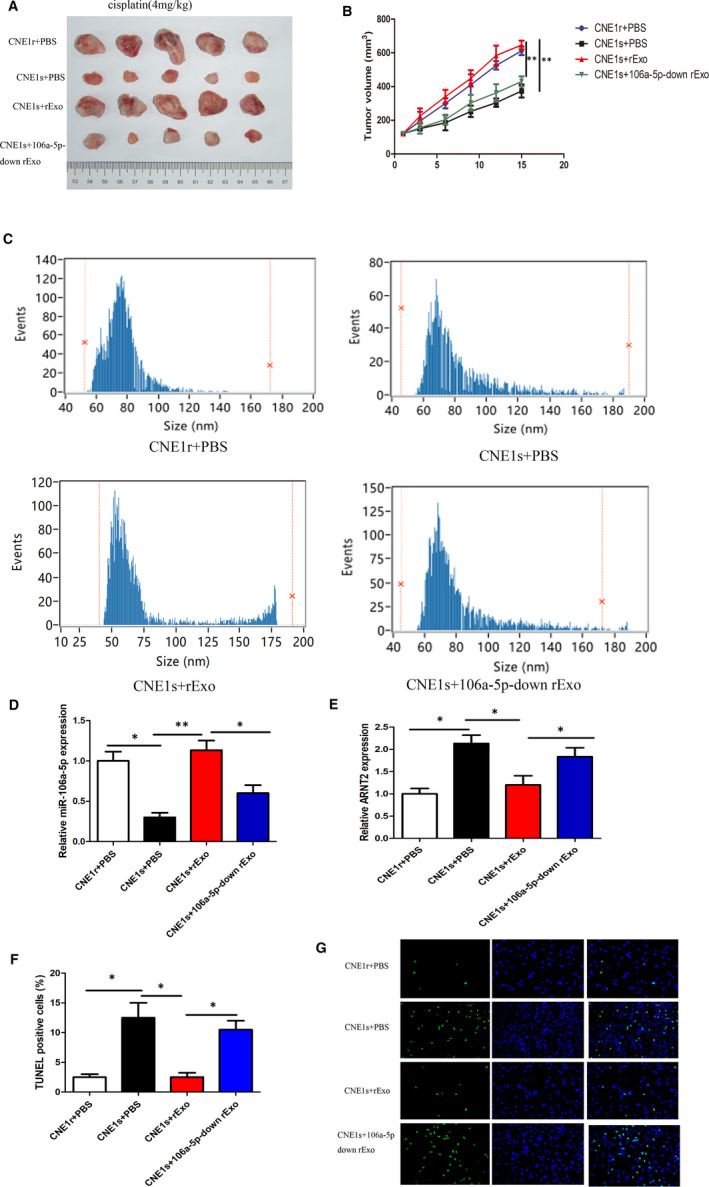
Exosomal miR‐106a‐5p promotes NPC cell cisplatin resistance *in vivo*. (A) Images of tumours removed from mice in each group CNE1r + PBS, CNE1s+ PBS, CNE1s + rExo and CNE1r + 106‐5p‐down rExo. (B) The tumour growth was quantified in tumours from CNE1s cells fed with resistant exosomes or 106a‐5p‐down exosomes after treatment with vehicle or cisplatin on the days indicated. (C) The size distribution of the isolated exosomes was measured by high‐sensitivity flow cytometry. (D) Relative expression of miR‐106a‐5p was measured by RT‐qPCR. (E) Relative expression of ARNT2 was measured by RT‐qPCR. (F) TUNEL staining of CNE1s cells injected into mice treated with cisplatin plus exosomal treatment. (G) Quantification of TUNEL‐positive cells from (F). In all experiments, bars represent mean ± SD for three replicates. *p < 0.05 and **p < 0.01

We further assessed the role of miR‐106a‐5p in promoting cisplatin resistance in an *in vivo* mouse model. Initially, we grouped the mice into four groups (*n* = 6) and injected intraperitoneally CNE1r cells with PBS (CNE1r + PBS) in the first group. In the second group, we injected CNE1s cells with PBS (CNE1s + PBS), and in the third group, we injected the mice with CNE1s cells cultured with rExo (CNE1s + rExo). Finally, the fourth group of mice was injected with CNE1s that were cultured with rExo, which were downregulated for miR‐106a‐5p (CNE1s + 106a‐5p‐down rExo). Mice were treated with cisplatin at 4 mg/kg of concentration. After 15 days, mice were killed, and tumours were extracted and imaged (Figure 7A). We also measured the tumour volume once every 3 days (Figure 7B). It was clear that mice in the CNE1r + PBS group had the largest tumours by 15 days, whereas mice in the CNE1s + PBS group had significantly smaller tumours. Furthermore, when CNE1s cells were cultured with rExo (CNE1s + rExo), the tumour size significantly increased similar to the CNE1r + PBS, confirming that indeed the resistance was transferred through rExo. Finally, the mouse group with CNE1s+106‐5p‐down rExo had smaller tumours similar to CNE1s + PBS (Figure 7A, B). Next, we isolated exosomes from the plasma of mice treated with different treatments. We found that there are differences in the number of exosomes in different treatment groups (Figure 7C). Further, we checked miR‐106a‐5p expression levels and observed that the CNE1r + PBS mouse group displayed high levels of miR‐106a‐5p. However, the CNE1s + PBS group displayed significantly low levels of miR‐106a‐5p levels when compared to the CNE1r + PBS group. However, when mice were injected with CNE1 cells cultured with rExo, we observed a significantly high level of miR‐106a‐5p. However, when mice were injected with CNE1s that had been treated with rExo downregulated for miR‐106a‐5p, miR‐106a‐5p was indeed downregulated in the samples (Figure 7D). In addition, the expression level of ARNT2 is shown in Figure 7 E. Finally, we performed TUNEL analysis to assess the apoptosis levels and observed that in the presence of cisplatin, mice injected with CNE1r+PBS displayed very less apoptosis. However, mice injected with CNE1s+PBS showed high sensitivity to cisplatin treatment, with increased apoptosis. Alternatively, mice injected with CNE1s + rExo showed significantly high resistance to cisplatin treatment and displayed little apoptosis. Mice with CNE1s + 106‐5p‐down rExo cells displayed sensitivity and high apoptosis in response to cisplatin, which indicated lack of miR‐106a‐5p decreases resistance to cisplatin treatment (Figure 7F, G). These *in vivo* results further confirmed our understanding of the role of miR‐106a‐5p on cisplatin resistance.

## 4 DISCUSSION

Exosomes play important roles in tumour progression and angiogenesis. Exosomes were found in higher concentrations in biological samples from patients with pancreatic, breast and ovarian cancers, indicating their importance as potential diagnostic markers.^26^ Exosomes carrying miR have recently been identified as important contributors to tumorigenesis. Antonyak et al^27^ observed that exosome carrying miR could contribute to tumour biogenesis and could also contribute to cell‐independent miR biogenesis, which is a unique phenomenon observed only in cancer cells exosomes.^27^ Recently, attention has been drawn to the contribution of exosomal miR to acquired chemotherapy resistance.^28^ The mechanism underlying exosomal miR function in drug resistance, on the other hand, is complex and relatively unknown. Exosomal miR‐106a‐5p was found to be highly upregulated in NPC in this study. Furthermore, we discovered that exosomal miR‐106a‐5p contributed to drug resistance in NPCs when treated with cisplatin.

Drug resistance is one of the key roadblocks in the advancement of chemotherapeutic treatment strategies, and cisplatin is among the most commonly used chemotherapeutic drugs.^28^ However, resistance to cisplatin treatment specifically to head and neck cancer has unfortunately become more prevalent in recent days.^29^ Specifically, the role of exosomal miRNA in drug resistance has been recently highlighted in many studies.^30^, ^31^ A study on lung cancer observed that miR‐100‐5p could regulate mTOR and thus confer resistance against cisplatin treatment.^31^ Interestingly, decreased exosomal miR‐100‐5p in the tumour microenvironment could downregulate the activation of mTOR by the endogenous miR‐100‐5p. This enabled increased expression of mTOR and resistance to cisplatin treatment. In other research on gastric^30^ and non–small‐cell lung cancer,^32^ exosomal miR‐21 and miR‐425‐3p were shown to play critical roles in cisplatin resistance by binding and controlling their downstream targets. Various mechanisms have been postulated regarding miR’s function in chemotherapeutic drug resistance, viz. drug efflux,^33^ metabolic reprogramming,^34^ DNA damage repair^35^ and apoptosis dysregulation.^30,32^ The above‐mentioned studies on gastric and non–small‐cell lung cancers indicated that exosomal miR‐21 and miR‐425‐3p regulate cisplatin resistance by activating the AKT pathway and, as a result, downregulating apoptosis in cancerous cells.^30,32^ Among the other miRs identified to play an important role in cisplatin resistance is miR‐196a in head and neck cancers. Evidentially, exosomal miR‐196a from cancer‐associated fibroblasts binds to CDKN11B and ING5 and confers cisplatin resistance via G1/S resistance and apoptosis regulation.^36^

miRs play key roles in cancer and many other disease models by binding to the 3’ UTR of their target genes. Studies have observed that indeed miRs competitively bind to specific regions upstream of the target gene's promoter region and either upregulate or downregulate their expression.^37^ In this study, we observed that miR‐106a‐5p binds to the 3'UTR of ARNT2 (aryl hydrocarbon receptor nuclear translocator 2) gene, a known transcriptional factor associated with adaptive responses against cellular stress.^38^ ARNT2 downregulation has recently been identified as a prognostic marker for poor survival in gastric cancer^39^ and increased cancer cell proliferation in oral squamous cell carcinoma.^38^ In this study, we also discovered that in NPCs, ARNT2 expression was downregulated, which leads to increased NPC cell proliferation, migration and invasion. This reduced ARNT2 expression could be reversed by lowering exosomal miR‐106a‐5p levels (Figure 4). A study on comprehensive miR profiling observed that indeed miR‐106a is among one of the most highly expressed miRs in head and neck cancer.^40^ However, this is the first study to confirm that indeed miR‐106a‐5p is highly upregulated and could be used as a prognostic marker for NPCs (Figure 1). Regarding drug resistance, a study identified that miR‐106a‐5p promotes 5‐Fluorouracil resistance in colorectal cancer.^41^ To our best knowledge, this is the first study to identify the role of miR‐106a‐5p on cisplatin resistance. Initially, we observed that miR‐106a‐5p levels increased in the resistant patients and specifically during the last cycles of cisplatin treatment (Figure 1). This clearly supported our findings of acquired resistance to cisplatin treatment. Surprisingly, circulating exosomes were found to be significantly higher in resistant patients and patients undergoing the final cycles of cisplatin treatment (Figure 1). We could mimic these observations in CNE1 cell models, where resistant cells had high levels of exosomal miRs than the sensitive cells (Figure 2). These results indicated that exosomal miR‐106a‐5p could be an ideal marker for predicting the prognosis of cisplatin treatment in NPC. Due to the heterogeneous genetic background of cells in a tumour microenvironment, cells could either be tumorous, non‐transformed and sensitive, or resistant to particular treatment.^42^ Cell‐cell communication through exosomes is a source of material transference inducing tumorigenesis or resistance in the tumour microenvironment.^28^ Exosomes containing miR‐106a‐5p from resistant CNE1 cells were found to confer resistance to cisplatin treatment in sensitive CNE1 cells in this study. Furthermore, inhibition of exosomal miR‐106a‐5p could significantly rescue this resistance by decreasing the cell viability and colony formation and increasing apoptosis during cisplatin treatment in NPC cells (Figure 3). As previously mentioned, miR‐106a‐5p regulated cisplatin resistance by binding to the 3’UTR of ARNT2, which we confirmed using luciferase activity assays. Furthermore, ARNT2 downregulation increased AKT phosphorylation, confirming that miR‐106a‐5p promotes cisplatin resistance by suppressing apoptosis in NPCs (Figures 5, 6). We subsequently also proved that indeed exosomes carrying miR‐106a‐5p confer resistance to cisplatin treatment using *in vivo* mouse models (Figure 7). Hence, this study discovered that exosomal miR‐106a‐5p promotes cisplatin resistance in NPC through the regulation of the ARNT2/AKT axis. miR‐106a‐5p could help to develop new diagnostic markers and treatment strategies for NPC.

## CONFLICT OF INTEREST

The authors confirm that there are no conflicts of interest to declare.

## AUTHOR CONTRIBUTIONS

**Jiaxing Li:** Investigation (equal); Software (equal); Writing‐original draft (equal). **Chaoquan Hu:** Formal analysis (equal); Investigation (equal); Writing‐original draft (equal). **Hui Cao:** Data curation (equal); Investigation (equal); Validation (equal). **Yu Zhang:** Software (equal); Validation (equal). **Yong Li:** Resources (equal); Software (equal). **Jing Hou:** Data curation (equal); Methodology (equal). **Limin Huang:** Conceptualization (equal); Writing‐review & editing (equal).

## Supporting information

Fig S1‐S2

## Data Availability

The data that support the findings of this study are available from the corresponding author upon reasonable request.
