# Supplementary material for: Exosomal transfer of miR‐106a‐5p contributes to cisplatin resistance and tumorigenesis in nasopharyngeal carcinoma
Source: J Cell Mol Med. 2021 Sep 1;25(19):9183–98. doi: 10.1111/jcmm.16801 (PMC8500979; doi:10.1111/jcmm.16801)
Supplement: Supplementary file 1 — Fig S1‐S2 [file JCMM-25-9183-s001.pdf]

Supplementary data

Exosomal transfer of miR-106a-5p contributes to cisplatin resistance and tumorigenesis in nasopharyngeal carcinoma

Jiaxing Li[a,1], Chaoquan Hu[b,1,*], Hui Chao[c], Yu Zhang[c], Yong Li[c], Jing Hou[c], Limin Huang[a,c*]

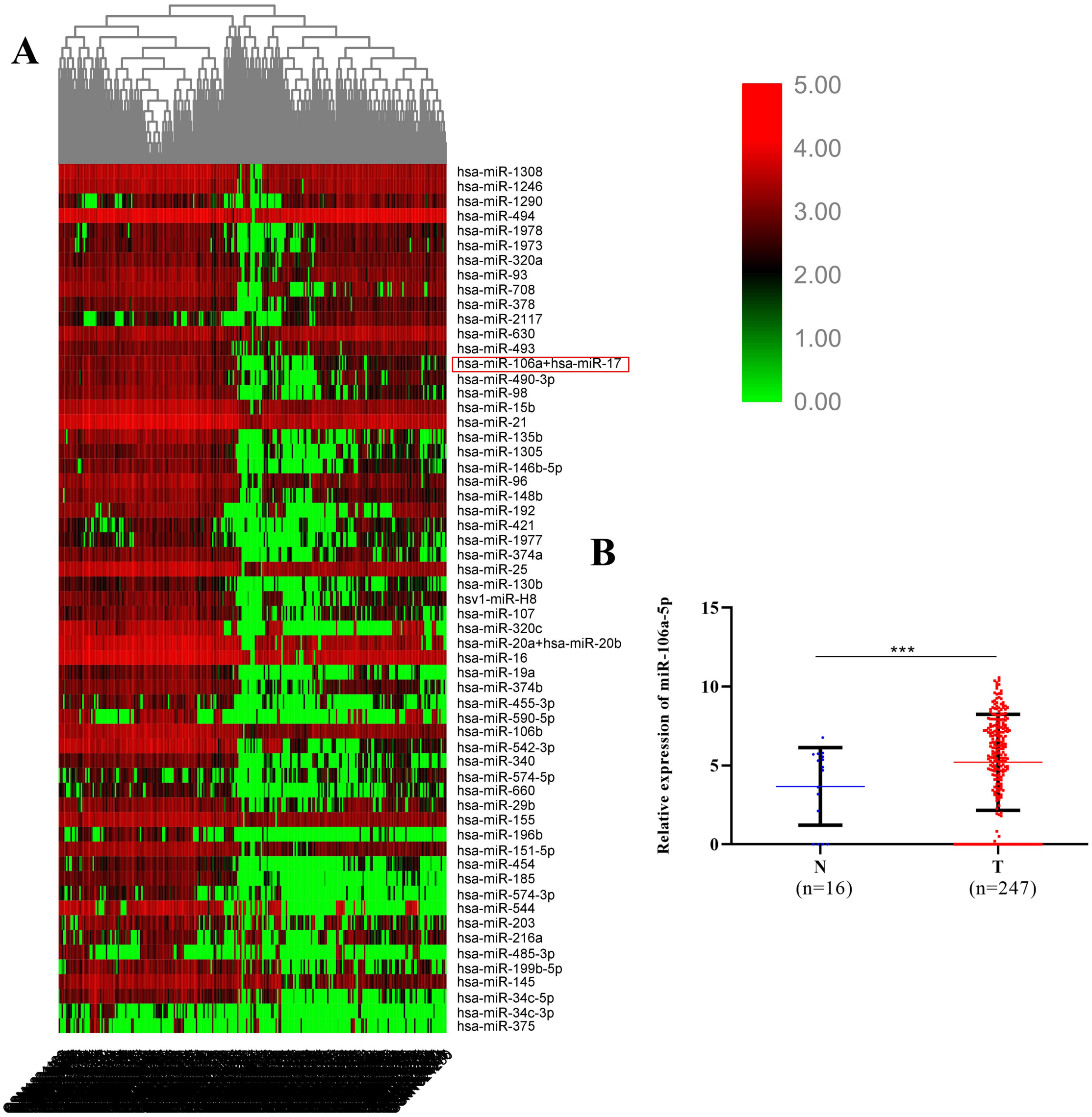

Figure S1. (A) Using the GEO chort database, different miRNA expression profiles for non-cancerous and NPC samples were examined (NO. GSE70970). (**B**) Levels of miR-106a-5p expression in GSE70970 chort data. Bars represent mean ± SD for three replicates.***P < 0.001.

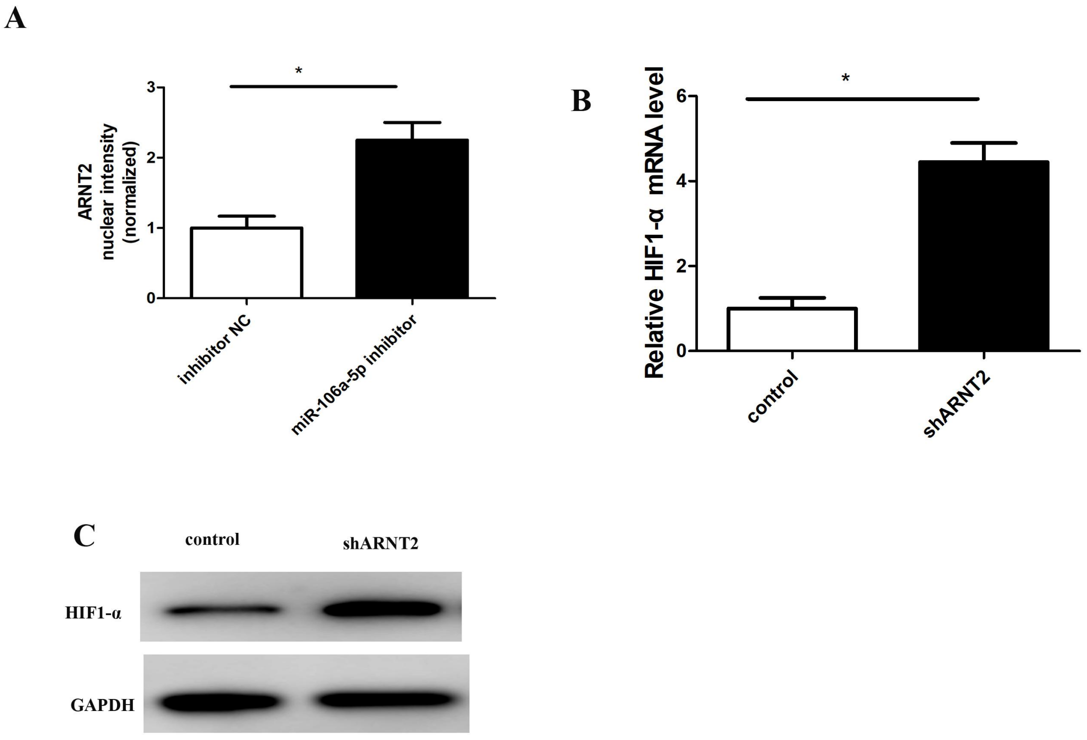

Figure S2. (**A**) Nuclear ARNT 2 expression in different treated cells. RT-PCR(**B**) and Western blot(**C**) were used to identify HIF1-α levels in different treated cells. Bars represent mean ± SD for three replicates.***P < 0.001.
